# The Combination of Galanin (1–15) and Escitalopram in Rats Suggests a New Strategy for Alcohol Use Disorder Comorbidity with Depression

**DOI:** 10.3390/biomedicines10020412

**Published:** 2022-02-09

**Authors:** Noelia Cantero-García, Antonio Flores-Burgess, David Ladrón de Guevara-Miranda, Antonia Serrano, Laura García-Durán, Araceli Puigcerver, Kjell Fuxe, José Ángel Narváez, Luis Javier Santín, Zaida Díaz-Cabiale, Carmelo Millón

**Affiliations:** 1 Facultad de Medicina, Instituto de Investigación Biomédica de Málaga, Campus de Teatinos s/n, Universidad de Málaga, 29071 Málaga, Spain; noeliacg@uma.es (N.C.-G.); afburgess@uma.es (A.F.-B.); lauragd_24@uma.es (L.G.-D.); bueno@uma.es (J.Á.N.); 2 Facultad de Psicología, Instituto de Investigación Biomédica de Málaga, Campus de Teatinos s/n, Universidad de Málaga, 29071 Málaga, Spain; david.lgm@uma.es (D.L.d.G.-M.); araceli@uma.es (A.P.); luis@uma.es (L.J.S.); 3 Unidad de Gestión Clínica de Salud Mental e Instituto de Investigación Biomédica de Málaga, Hospital Regional Universitario de Málaga, 29010 Málaga, Spain; antonia.serrano@ibima.eu; 4 Department of Neuroscience, Karolinska Institute, 17177 Stockholm, Sweden; kjell.Fuxe@ki.se

**Keywords:** Galanin (1–15), escitalopram, depression, alcohol

## Abstract

Alcohol use disorder (AUD) is highly prevalent, and over 50% of AUD patients also suffer major depressive disorders. Selective 5-HT reuptake inhibitors (SSRIs) can reduce rodent ethanol drinking but exert modest clinical efficacy in alcoholic individuals. Finding new pharmacological strategies that could modulate alcohol consumption and depression is necessary. We have analyzed the effect of Galanin (1–15) [GAL(1–15)] on escitalopram (ESC)-mediated effect in alcohol consumption using the alcohol self-administration test, the nuclei involved in the effect, and whether GAL(1–15) + ESC modulated the response in despair or anxiety tests in animals under chronic alcohol intake. GAL(1–15) + ESC combination substantially reduced alcohol intake in the alcohol self-administration test and, moreover, enhanced the reduction of reward capacity of ESC on different reinforcers such as sucrose or saccharine. GAL(1–15) + ESC coadministration significantly decreases the number of C-Fos-IR TH cell bodies in the VTA, and PCA analysis suggests that one functional network, including VTA, RMTg and DR, is involved in these effects. Significantly in rats with chronic alcohol consumption, GAL(1–15) reversed adverse ESC-mediated effects in the depression-related behavioural test and forced swimming test. The results open up the possibility of using GAL(1–15) in combination with the SSRI Escitalopram as a novel strategy in AUD comorbidity with depression.

## 1. Introduction

Alcohol use disorder (AUD) is a highly prevalent psychiatric disorder, and over 50% of treated AUD patients also suffer from other psychiatric disorders, including major depressive disorders [1]. The rate of AUD and depression comorbidity have been assessed in epidemiologic studies that linked AUD to 3.7-fold higher odds of experiencing a depressive episode in the prior year [2], and individuals with a lifetime diagnosis of major depression to a 1.3-fold increased risk of AUD [3]. Furthermore, these studies found that major depression worsened the symptoms of AUD and vice versa [4], which may explain why people with comorbid depression and AUD are at greater risk for suicide [5,6].

Both psychiatric disorders, AUD and depression, are associated with dysregulated brain monoaminergic systems including the serotoninergic (5-HT) system. A link between serotoninergic neurotransmission and alcohol intake is suggested based on preclinical studies of alcohol administration in animals and humans, neuropathological examination of alcohol-preferring animals, and application of pharmacological probes that affect the 5-HT system [7,8].

Thus, selective 5-HT reuptake inhibitors (SSRIs) can reduce rodent ethanol drinking and exert modest clinical efficacy in some subpopulations of alcoholic individuals [9,10]. Serotonin transporter knockout mice showed lower alcohol consumption in a free-choice paradigm, and this model demonstrated that the inhibitory effect of fluoxetine, the oldest and best-studied SSRI, on alcohol intake is the consequence of its direct interaction with the transporter responsible for 5-HT reuptake [10,11]. Treatment with other SSRIs such as citalopram also reduced alcohol consumption in alcohol-naïve rats and rats with high preference for intake of alcohol [12,13].

Although clinical studies found SSRIs improved depressed mood and decreased alcohol use in some alcohol-dependent individuals [14], the efficacy of SSRI in AUD and depression comorbidity patients is unclear [8,15,16]. Antidepressants had positive effects on specific relevant outcomes related to depression and alcohol use but not on others [16]. It is, therefore, of great importance to find new pharmacological strategies that could modulate alcohol consumption and depression. Escitalopram (ESC), a pure S-enantiomer of citalopram, 100-fold more potent than the R-enantiomer in inhibiting 5-HT reuptake [17], is currently one of the most widely used SSRIs [18]. The combination of ESC with other compounds, such as acamprosate or aripiprazole in AUD patients with comorbid major depression, showed a significant improvement in depressive symptoms despite ongoing drinking [19,20,21].

We have described that the neuropeptide Galanin (1–15) [GAL(1–15)] induces strong depression and anxiogenic-like effects [22,23] and also a strong anhedonia-like phenotype, a key symptom of depression [24], acting through GALR1-GALR2 heteroreceptor complexes in the CNS [23,25]. However, GAL(1–15) is able to enhance the antidepressant effects induced by the 5-HT1AR agonist 8-OH-DPAT in the forced swimming test (FST) [26], which involves alterations in both the binding characteristics and mRNA levels of 5-HT1AR in the dorsal hippocampus and dorsal raphe [26].

Moreover, GAL(1–15) combined with SSRIs could improve their effectiveness in treating depression symptoms. Thus, we observed in rats that GAL(1–15) enhanced the antidepressant effects induced by fluoxetine in tests related to despair and anhedonic behaviour. This GAL(1–15) effect was also observed in an olfactory bulbectomized rat (OBX) model of depression [27,28]. GAL(1–15) also enhanced the antidepressant–like effects induced by ESC in OBX rats, and 5HT1AR significantly participates in the GAL(1–15)/ESC interaction [29]. Two functional networks were involved in the effects; one of them includes the lateral (LHb) and medial (mHB) habenula, dorsal raphe (DR) and ventral tegmental area (VTA), and the other includes the dentate gyrus (DG) and prefrontal cortex (PFC) [29].

Since GAL(1–15) also induces a substantial reduction in preference and voluntary alcohol consumption in rats [30] with involvement of the dopaminergic mesolimbic system, which is critical for the reward system [24,30], the combination of GAL(1–15) with ESC could be proposed as an effective treatment for AUD patients with comorbid major depression. However, we need additional information from animal models on the effect of this combination in alcohol consumption and in behavioural tests related to despair in animals under chronic alcohol intake.

In the current study, we analyzed the effect of GAL(1–15) on ESC-mediated effects in alcohol consumption using the alcohol self-administration test, a widely accepted approach to assess reward-seeking behaviours. Additionally, to study if the combination GAL(1–15) and ESC modulate the reward system induced by reinforcers different from alcohol, we analyzed this combination in two other rewarding tests: the sucrose preference test (SPT) and the saccharine self-administration test. Moreover, in animals under chronic alcohol intake by self-administration, we studied whether GAL(1–15) + ESC combination modulated the response in behavioural tests related to despair (FST and TST) or anxiety (open field test and elevated plus-maze). To investigate the brain areas involved in GAL(1–15) + ESC effects in alcohol consumption, we analyzed the immunohistochemistry of immediate early gene C-Fos as an indirect marker of neural activity. We studied the expression of C-Fos expression after the administration of GAL(1–15) + ESC in several nuclei involved in depression and reward-seeking behaviour—lateral (LHb) and medial (mHb) habenula, nucleus accumbens (NAc), prefrontal cortex (CPF) and the rostromedial tegmental nucleus (RMTg)—and performed double immunohistochemical staining of 5-hydroxytryptamine (5-HT) and C-Fos or tyrosine hydroxylase (TH) and C-Fos to study the specific cell activation in the dorsal raphe (DR) and ventral tegmental area (VTA), respectively. Additionally, we assessed the brain circuits using principal component analysis (PCA) in the multivariate analysis used to understand brain functional organization.

## 2. Material and Methods

### 2.1. Animals

Male Sprague Dawley rats (body weight 225–250 g) were obtained from criffa and maintained in a humidity-controlled and temperature-controlled (20–22 °C) room. During the entire protocol, rats were maintained on a 12-h reversed light/dark cycle (lights off at 9 a.m.). All animal experimentation was conducted in accordance with the University of Málaga Guidelines for the Care and Use of Laboratory Animals (Ethic Code: 22/05/2017/066).

Detailed descriptions are available in the supplementary information on the animal controlled-conditions, surgical preparation and administration of substances and drugs.

### 2.2. Experimental Design 

**Figure 1:**
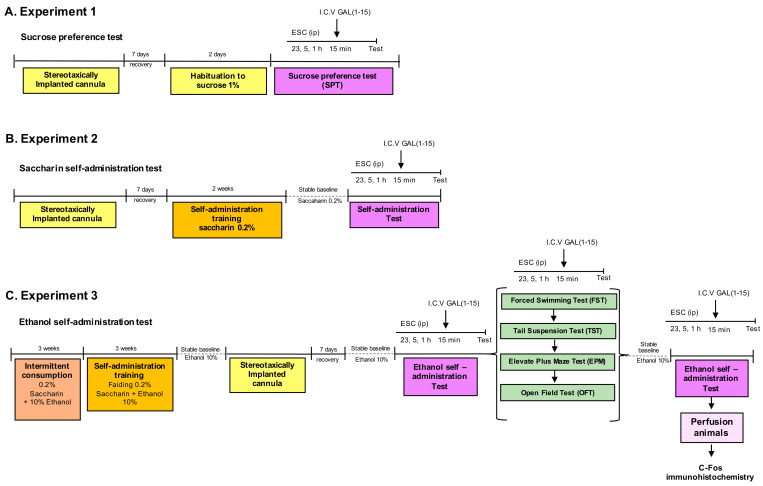
Diagram of the complete experimental schedule. We analyzed the effect of the combination of Escitalopram (ESC) and Galanin (1–15) [GAL(1–15)] in the sucrose preference test (SPT) (**A**) and the saccharin self-administration test (**B**). In (**C**), we studied the effect of the combination of ESC and GAL(1–15) in the ethanol self-administration test, forced swimming test, tail suspension test, elevated plus maze and open field test.

Three experimental procedures were carried out. The scheme of the experimental design is shown in Figure 1.

#### 2.2.1. Experiment 1

We analyzed the effect of the combination of ESC and GAL(1–15) in the sucrose preference test.

In the experiment, first, a dose-response curve of ESC was performed. Groups of rats received three separate intraperitoneal injections of ESC 23, 5 and 1 h before the beginning of the tests at the doses of 7.5 mg/kg or 10 mg/kg or vehicle. 

Once we determined the ESC effect, we studied the effect of the coadministration of ESC and GAL(1–15) on sucrose intake and in the sucrose preference test. For this, groups of rats received three separate intraperitoneal injections of vehicle or ESC (10 mg/kg) 23, 5 and 1 h before the beginning of the test and one intracerebroventricular (icv) injection of GAL(1–15) 1 nmol or aCSF 15 min before the test. The sucrose intake and preference were measured for 2 h, beginning 15 min after administering GAL(1–15) or aCSF.

#### 2.2.2. Experiment 2

We analyzed the effect of the combination of ESC and GAL(1–15) in the saccharin self-administration test.

First, a dose-response curve of ESC was calculated. Groups of rats received three separate intraperitoneal injections of ESC 23, 5 and 1 h before the tests at the doses of 2.5 mg/kg; 5 mg/kg; and 7.5 mg/kg or vehicle. 

Once we determined the ESC effect, we studied the effect of the coadministration of ESC and GAL(1–15) in the saccharin self-administration test. 

Groups of rats received three separate intraperitoneal injections of vehicle or ESC (2.5 mg/kg or 7.5 mg/kg) 23, 5 and 1 h before the beginning of the test and one icv injection of GAL(1–15) 1 nmol or aCSF 15 min before the test.

#### 2.2.3. Experiment 3

First, we analyzed the combination of GAL(1–15) and ESC in rats with chronic alcohol consumption by self-administration. Afterwards, we studied the combined ESC + GAL(1–15) effect in several behavioural tests for depression and anxiety in rats with chronic alcohol consumption (daily alcohol self-administration continued for 2 months).The general scheme of the experimental design is shown in Figure 1C.

Groups of rats received three separate intraperitoneal injections of vehicle or ESC (2.5 mg/kg) 23, 5 and 1 h before the beginning of the test and one icv injection of GAL(1–15) 0.3 nmol or aCSF 15 min before the test. The number of alcohol reinforcement and number of active lever presses were assessed with the self-administration test.

In the second series of experiments, the rats with chronic alcohol consumption received three separate intraperitoneal injections of vehicle or ESC (7.5 mg/kg) 23, 5 and 1 h before the beginning of the tests and one icv injection of GAL(1–15) 1 nmol or aCSF 15 min before the depression-related tests FST and TST. The dose of ESC 7.5 mg/kg was based on our previous works in FST and TST [31].

Finally, the rats with chronic alcohol consumption received three separate intraperitoneal injections of ESC 23, 5 and 1 h before the beginning of the tests at the doses of 2.5 mg/kg or vehicle and one icv injection of GAL(1–15) 0.3 nmol or aCSF 15 min before the test to evaluate the effects in the anxiety-related tests EPM and OFT.

The doses of GAL(1–15) employed in the rewarding tests and the anxiety and depression-related tests were based on previous studies [22,24,29]. The doses of GAL(1–15) in the ethanol self-administration test were based on a GAL(1–15) dose-response curve in this test (data not shown).

#### 2.2.4. Behavioural Assessment (SPT)

Reward capacity was assessed using the SPT, performed as described previously [24]. Briefly, on the testing day, rats were allowed free access to two bottles: one containing 1% (*w*/*v*) sucrose solution and the other containing tap water. After 2 h, the bottles were weighed to calculate the sucrose intake (g/kg) and sucrose preference [sucrose preference = (sucrose consumption/(water + sucrose consumption) × 100], which reflected the rats’ anhedonia levels.

#### 2.2.5. Saccharin Self-Administration 

Reward capacity was assessed using the self-administration test, performed as described previously [24]. Briefly, rats were placed on a water restriction schedule for 2–4 days to facilitate training of lever pressing. The rats were trained to self-administer saccharin 0.2% (*w*/*v*) in 30-min daily sessions for 2 weeks on a fixed ratio 1 schedule of reinforcement in which each response resulted in the delivery of 0.1 mL of fluid. One lever was paired with the delivery of saccharin as a reward (active lever), whereas the other lever was paired with no reward (inactive lever). At this point, saccharin self-administration training continued until the animals reached stable baseline responding. During the 30 min test sessions, the responses on the active lever and number of saccharin reinforcements were recorded (see the Supplementary Materials for details).

#### 2.2.6. Ethanol Self-Administration Test

Ethanol consumption was assessed using the self-administration test, performed as described previously [24,32] with minor modifications (see the supplementary information for details). Briefly, rats were exposed to intermittent consumption of 0.2% saccharin and 10% ethanol for 3 weeks. After that, rats were placed on a water restriction schedule for 2–4 days to facilitate training of lever pressing. The rats were trained to self-administer saccharin 0.2% (*w*/*v*) and 10% ethanol (*v*/*v*) in 30 min daily sessions for 2 weeks on a fixed ratio 1 schedule of reinforcement in which each response resulted in the delivery of 0.1 mL of fluid. One lever was paired with the delivery of ethanol as a reward (active lever), whereas the other lever was paired with no reward (inactive lever). At this point, ethanol self-administration training continued until the animals reached a stable level of 10% ethanol responding. During the 30 min test sessions, the responses on the active lever and number of alcohol reinforcements were recorded (see the Supplementary Materials for details).

#### 2.2.7. Forced Swimming Test (FST)

Depressive behaviour was assessed using the FST, performed as described previously [23,26,28]. Briefly, two swimming sessions were conducted: a 15 min pre-test followed 24 h later by a 5 min test. Animals were individually placed in a vertical glass cylinder of 20 cm diameter containing water (25 °C) to a height of 30 cm. The total durations of immobility and swimming behaviour were recorded during the second 5 min test. 

#### 2.2.8. Tail Suspension Test (TST)

Depressive behaviour was assessed using the TST, performed as described previously [23,29]. Briefly, rats were hung upside down using an adhesive tape to fix the tail to a rope through an eyebolt at 60 cm. The animal was considered immobile when it was not making any movements of struggling, attempting to catch the adhesive tape, body torsions, or jerks time. The total durations of immobility and mobility behaviour were recorded during the second 5 min test.

#### 2.2.9. Elevated Plus Maze (EPM)

Anxiety behaviour was assessed using the EPM as previously described [33,34]. Briefly, rats were placed on the central platform facing an open arm and allowed to explore the maze for 5 min. The time in open arms and entries to the center were analyzed using the video-tracking software EthovisionXT (see the Supplementary Materials for details).

#### 2.2.10. Open Field Test (OFT)

Anxiety behaviour was assessed using the OFT as previously described [23]. Briefly, the rats were placed in the open field (100 × 100 × 50 cm) and allowed to explore freely for 5 min. Total time spent and interior square inputs were analyzed using EthovisionXT video tracking software.

### 2.3. Immunohistochemistry

Groups of rats received three separate intraperitoneal injections of vehicle or ESC (2.5 mg/kg) and one icv injection of GAL(1–15) 0.3 nmol or aCSF. The dose of GAL(1–15) 0.3 nmol used was 10 times lower than the effective dose of GAL(1–15) in C-Fos immunohistochemistry [30,35].

Ninety min after drug administration, rats were anaesthetized with sodium pentobarbital (Mebumal; 100 mg/kg body weight, i.p.) and intracardially perfused with 200 mL isotonic ice-cold saline phosphate buffer followed by 200 mL of fixation fluid 4% paraformaldehyde (*w*/*v*) in saline 0.1 M sodium PB (PBS), pH 7.4). The brains were removed, postfixed for 12 h in the same fixative, and cryoprotected in sucrose (30% at 4 °C). Brainstem 30 μm coronal sections were obtained on a cryostat. The sections were sequentially incubated with primary antibodies anti-C-Fos (mouse polyclonal antibody 1/1200, sc-271243, Santa Cruz Biotech, Dallas, TX, USA); anti-5-HT (rabbit monoclonal antibody 1/20,000, 20,080, INCSTAR, Stillwater, MN, USA); anti-TH (mouse monoclonal antibody 1/2500, T1299, Sigma, St. Louis, MO, USA) and anti-pCREB (rabbit polyclonal antibody 1/500, 06-519 Millipore, Burlington, MA, USA) (see Supplementary Materials for further details).

The immunoreactivity was analyzed in dorsal raphe (RD), rostromedial tegmental nucleus (RMTg), lateral habenula (LHb), medial habenula (MHb), ventral tegmental area (VTA), nucleus accumbens (NAc) and prefrontal cortex (CPF). Double immunohistochemical staining of 5-HT and C-Fos-IR or TH and C-Fos-IR were used to study the specific cell activation in the DR and VTA.

In the immunohistochemical assays, the cell count was normalized to the total area.

### 2.4. Statistical Analysis

Data are presented as the mean ± standard error of the mean, and sample numbers (*n*) are indicated in figure legends. All data were analyzed using GraphPad PRISM 8.0 (GraphPad Software, San Diego, CA, USA). For comparing more than two groups, one-way analysis of variance (ANOVA) was performed. Fisher’s least significant difference (LSD) comparison post-test was performed only when the F ratio in the one-way ANOVA was statistically significant. Differences were considered statistically significant at *p* < 0.05 (* *p* < 0.05, ** *p* < 0.01, *** *p* < 0.001). 

A PCA with varimax rotation was also performed to extract the independent dimensions (i.e., factors) from the C-Fos IR data. Eigenvalue > 1 was chosen as criterion for component extraction and a factor score (i.e., a standardized value indicating the relative position of each animal in each factor) was computed by the regression method (SPPS Statistics 20, IBM Corporation, Armonk, NY, USA). Only measures with a saturation greater than 0.5 in absolute value were included in a factor. The ability of each factor to predict ethanol-seeking behaviour was tested by Pearson’s correlations between the factorial score and the rat-score behaviour (see the Supplementary Materials for details).

## 3. Results

### 3.1. Behavioural Effects of the Combination of GAL(1–15) and ESC in SPT and Saccharine Self-Administration Test in Rats

#### 3.1.1. Dose-Response Curve of ESC in SPT and Saccharine Self-Administration Test

In SPT, ESC (7.5 mg/kg) induced a significant reduction in sucrose intake (one-way ANOVA, F_2,25_ = 4.46, *p* = 0.02, Fisher’s LSD post hoc: *p* < 0.01) and in sucrose preference (one-way ANOVA, F_2,25_ = 4.65, *p* = 0.01, Fisher´s LSD post hoc: *p* < 0.05, *p* < 0.01) (Table S1) compared with control animals. However, ESC at 10 mg/kg lacked an effect on sucrose intake or sucrose preference compared with controls animals (Table S1).

In the saccharine self-administration test (Figure S1), ESC at the doses of 5 mg/kg (*p* < 0.05) and 7.5 mg/kg (*p* < 0.001) significantly decreased the number of saccharine reinforcements (one-way ANOVA, F_3,46_ = 5.05, *p* = 0.004, Fisher´s LSD post hoc: *p* < 0.05, *p* < 0.001; Figure S1B) by 60 and 75%, respectively, and the number of active lever presses (one-way ANOVA, F_3,46_ = 4.89, *p* = 0.004, Fisher´s LSD post hoc: *p* < 0.05, *p* < 0.001; Figure S1B) compared with the control group. No effects were observed on the number of inactive lever presses at these doses. ESC at a dose of 2.5 mg/kg lacked any effect in the parameters analyzed in the saccharine self-administration test (Figure S1B). 

#### 3.1.2. GAL(1–15) Enhances the Reduction of Reward Capacity of ESC in the SPT and in the Saccharine Self-Administration Test in Rats

**Figure 2:**
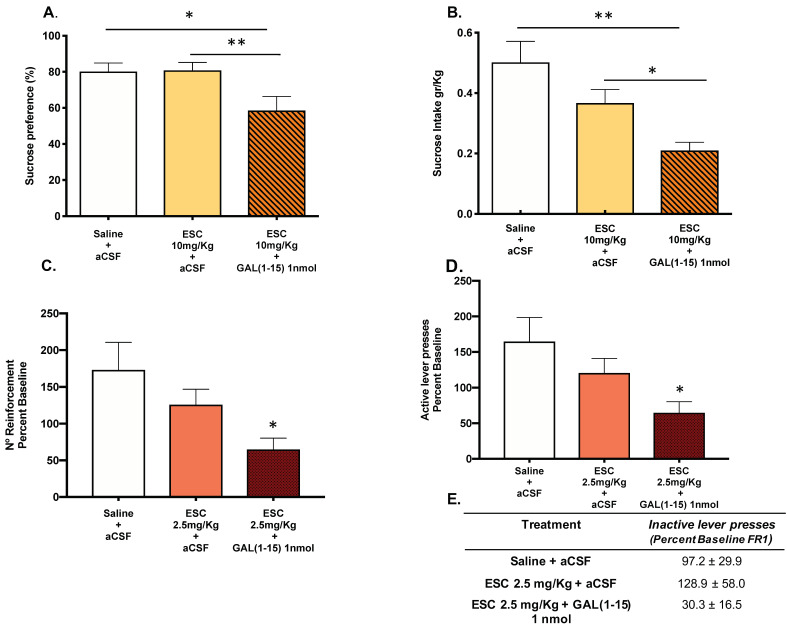
Effect of the administration of Galanin (1–15) [GAL(1–15)] and Escitalopram (ESC) in the sucrose preference test (SPT) and the saccharin self-administration test. ESC was administrated intraperitoneal (ip) 23, 5 and 1 h before the test and GAL(1–15) 1 nmol or artificial cerebrospinal fluid (aCSF) was administered icv 15 min before the test. Saline + aCSF injected rats were used as the control group (*n* = 7–10 animals/group). SPT (**A**,**B**): Vertical bars represent the mean ± standard error of the mean of sucrose intake (g/kg) and preference (percentage). * *p* < 0.05; ** *p* < 0.01, according to one way ANOVA followed by Fisher multiple comparison test. The saccharin self-administration test (**C**,**D**): Vertical bars represent mean ± standard error of the mean number of saccharin reinforcements and active lever presses according to the percent baseline during the test period. * *p* < 0.05 vs. saline + aCSF, according to one-way ANOVA followed by Fisher multiple comparison test. (**E**): Data represent mean ± standard error of the mean of inactive lever presses according to percent baseline during the test period. There were no differences according to a one-way analysis of variance (ANOVA) between the experimental groups.

The coadministration of ESC (10 mg/kg) and the icv injection of GAL(1–15) (1 nmol) induced a decrease in sucrose intake (one-way ANOVA, F_2,23_ = 6.89, *p* = 0.004, Fisher´s LSD post hoc: *p* < 0.05, *p* < 0.01; Figure 2A) and in sucrose preference (one-way ANOVA, F_2,23_ = 4.80, *p* = 0.01, Fisher´s LSD post hoc: *p* < 0.05, *p* < 0.01; Figure 2B) compared with the ESC (10 mg/kg) group in the sucrose preference test, suggesting that GAL(1–15) enhanced the reduction of reward capacity mediated by ESC in this test. 

In the saccharine self-administration test, the coadministration of threshold doses of GAL(1–15) (1 nmol) and ESC (2.5 mg/kg) induced a strong reduction in the reward capacity of saccharine. Thus, a significant decrease appeared in the number of reinforcements of saccharine self-administration (one-way ANOVA, F_2,21_ = 3.85, *p* = 0.03 Fisher´s LSD post hoc: *p* < 0.05; Figure 2C) and in number of active lever presses (one-way ANOVA, F_2,21_ = 3.86, *p* = 0.04, Fisher´s LSD post hoc: *p* < 0.05; Figure 2D). Although the coadministration of GAL(1–15) + ESC lacked effects on the number of inactive lever presses (Figure 2E), the high variance showed by the animals could contribute to it.

The dose of ESC (7.5 mg/kg) induced a significant reduction in the number of reinforcements (one-way ANOVA, F_2,20_ = 10.2, *p* = 0.0009, Fisher´s LSD post hoc: *p* < 0.001; Figure S1C) and active lever presses (one-way ANOVA, F_2,20_ = 9.25, *p* = 0.001, Fisher´s LSD post hoc: *p* < 0.01; Figure S1D). The coadministration of this effective dose of ESC (7.5 mg/kg) and GAL(1–15) (1 nmol), but not ESC alone, also induced a strongly significant reduction in the number of reinforcements and active lever presses compared with control animals (Fisher’s LSD post hoc: *p* < 0.01, *p* < 0.001; Figure S1).

GAL(1–15) 1 nmol alone had no effect on either the number of saccharine reinforcements or the number of active lever presses in saccharine self-administration [24].

### 3.2. Behavioural Effects of the Combination of GAL(1–15) and ESC in Rats with Chronic Alcohol Consumption by Self-Administration

#### 3.2.1. The Combination of GAL(1–15) and ESC Induced a Substantial Reduction of Alcohol Intake in the Ethanol Self-Administration Test

**Figure 3:**
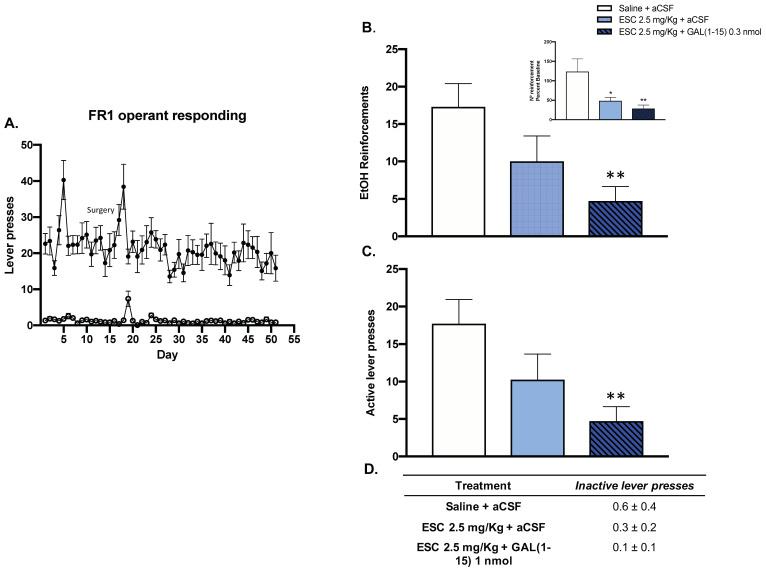
Effect of the administration of Galanin (1–15) [GAL(1–15)] and Escitalopram (ESC) in the ethanol self-administration Test. ESC was administrated intraperitoneally (ip) 23, 5 and 1 h before the test, and GAL(1–15) 0.3 nmol or aCSF was administered i.c.v 15 min before the test. Saline + aCSF, injected rats were used as control group (*n* = 7–8 animals/group). (**A**) Animals displayed a consistent preference for 10% *v*/*v* ethanol (active lever) over no reward (inactive lever) during the FR1 operant responding phase. (**B**,**C**) Vertical bars represent mean ± standard error of the mean of the number of ethanol reinforcements and active lever presses during the test period. In the figure, the data are represented according to percent baseline during the test period. * *p* < 0.05 vs. saline + aCSF, ** *p* < 0.01 vs. saline + aCSF, according to one-way analysis of variance (ANOVA) followed by Fisher’s least significance difference test. (**D**) Data represent mean ± standard error of the mean of inactive lever presses during the test period. There were no differences according to a one-way analysis of variance (ANOVA) between the experimental groups.

In the alcohol self-administration test, animals displayed a consistent preference for 10% *v*/*v* ethanol (active lever) over no reward (inactive lever) during the FR1 operant responding phase (Figure 3A).

In this test, the threshold dose of GAL(1–15) (0.3 nmol) enhanced the reduction of alcohol intake mediated by ESC (2.5 mg/kg) in rats. Icv GAL(1–15) decreased the number of alcohol reinforcements, by 50% (one-way ANOVA, F_2,19_ = 4.35, *p* = 0.02, Fisher´s LSD post hoc: *p* < 0.01 Figure 3B), and the number of active lever presses, by around 50% (one-way ANOVA, F_2,19_ = 4.50, *p* = 0.02, Fisher´s LSD post hoc: *p* < 0.01) (Figure 3C), induced by ESC. However, the combination of GAL(1–15) and ESC had no effect on the number of inactive lever presses in the alcohol self-administration test (Figure 3D).

A dose-response curve of GAL(1–15) in the alcohol self-administration test was compiled. GAL(1–15) 0.3 nmol is a threshold dose since it lacked an effect on the number of reinforcements or number of lever presses in the ethanol self-administration test (data not shown).

#### 3.2.2. In Depression Tests Related to Despair, GAL(1–15) Reversed the Adverse ESC-Mediated Effects in Rats with Alcohol Consumption by Self-Administration

**Figure 4:**
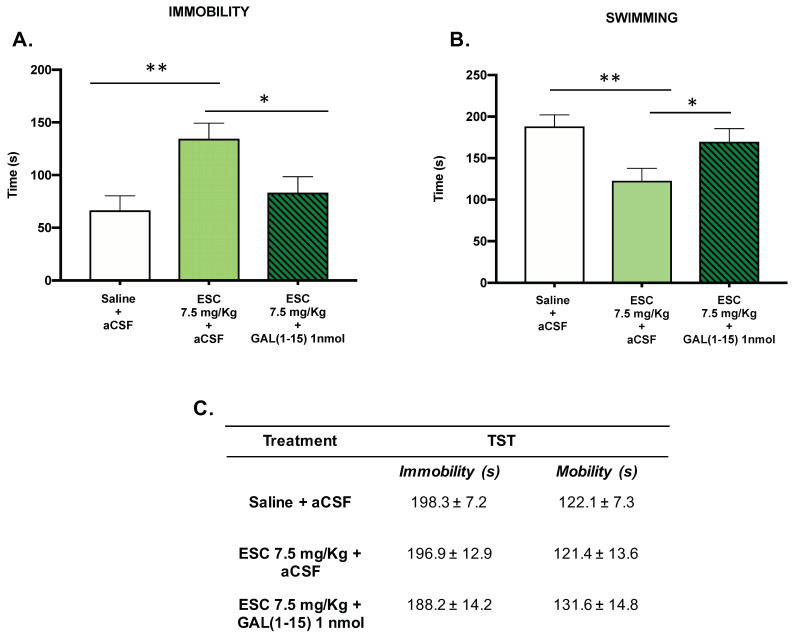
Effect of the administration of Galanin (1–15) [GAL(1–15)] and Escitalopram (ESC) in the forced swimming test (FST) and the tail suspension test (TST). ESC was administrated intraperitoneal (ip) 23, 5 and 1 h before the test, and GAL(1–15) 1 nmol or artificial cerebrospinal fluid (aCSF) was administered i.c.v 15 min before the test. Saline + aCSF injected rats were used as the control group (*n* = 7–8 animals/group). FST (**A**,**B**): Vertical bars represent the mean ± standard error of the mean of immobility time and swimming time in FST during the test period. * *p* < 0.05, ** *p* < 0.01, according to a one-way analysis of variance (ANOVA) followed by Fisher’s least significance difference test. TST (**C**): Data represent mean ± standard error of the mean of immobility time and mobility time during the test period. There were no differences according to a one-way analysis of variance (ANOVA) between the experimental groups.

In the FST, the subchronic administration of ESC (7.5 mg/kg) in animals with ethanol intake by self-administration induced a significant increase in the immobility time (one-way ANOVA, F_2,19_ = 5.86, *p* = 0.01, Fisher´s LSD post hoc: *p* < 0.01 Figure 4A) and a decrease in the swimming time (one-way ANOVA, F_2,19_ = 5.21, *p* = 0.01, Fisher’s LSD post hoc: *p* < 0.01; Figure 4B) compared with the control group, suggesting an adverse effect of ESC in this test.

The coadministration of GAL(1–15) (1 nmol) and ESC (7.5 mg/kg) reversed the effect of ESC administration, as the animals cotreated with GAL(1–15) + ESC showed a statistically significant decrease in immobility (Fisher’s LSD post hoc: *p* < 0.05) (Figure 4A) and an increase in swimming (Fisher’s LSD post hoc: *p* < 0.05)(Figure 4B) compared with the ESC group.

However, in the TST, both the administration of ESC (7.5 mg/kg) alone and the coadministration of GAL(1–15) (1 nmol) and ESC (7.5 mg/kg) did not show significant differences in immobility or mobility time.

In the anxiety tests, no significant effects were found after the coadministration of GAL(1–15) (0.3 nmol) and ESC (2.5 mg/kg) (Table 1) in either the EPM or in the OFT in chronic ethanol exposure rats.

### 3.3. C-Fos Immunohistochemistry Study of GAL(1–15) and ESC Interaction in Rats with Chronic Alcohol Consumption

**Figure 5:**
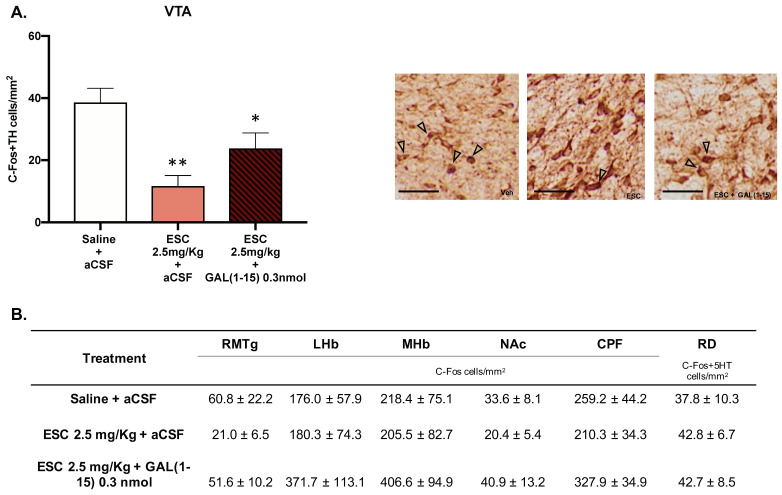
Effect of the administration of Galanin (1–15) [GAL(1–15)] and Escitalopram (ESC) on C-Fos expression. Data represent mean ± SEM of C-Fos cells/mm^2^ × 10 in the rostromedial tegmental nucleus (RMTg), lateral habenula (LHb), medial habenula (mHb), nucleus accumbens (NAc) and prefrontal cortex (PFC). The mean ± SEM of C-Fos + 5-HT in DR and C-Fos + TH cells/mm^2^ in the ventral tegmental area (VTA) is also shown (*n* = 4–5 animals/group). (**A**) * *p* < 0.05 vs. saline + aCSF; ** *p* < 0.01 vs. saline + aCSF, according to a one-way analysis of variance (ANOVA) followed by Fisher’s least significance difference test. In subfigure (**A**), a representative photomicrograph illustrating the different treatments is also shown. Scale bar = 100 μm. (**B**) There were no differences according to a one-way analysis of variance (ANOVA) between the experimental groups.

We analyzed C-Fos IR 90 min after the coadministration of GAL(1–15) (0.3 nmol) and ESC (2.5 mg/kg) in several nuclei involved in depression and AUD—RMTg, LHb, mHb, NAc, PFC—and by double immunohistochemical staining of 5HT/C-Fos in DR or TH/C-Fos in VTA (Figure 5A,B). 

As seen in Figure 5, in the VTA, the number of C-Fos IR TH cell bodies after icv GAL(1–15) + ESC (one-way ANOVA, F_2,11_ = 9.03, *p* = 0.004, Fisher´s LSD post hoc: *p* < 0.05) and ESC (Fisher´s LSD post hoc: *p* < 0.01) was significantly decreased in comparation with C-Fos IR TH cell bodies in the control group (Figure 5A). 

No significative effects were observed in the rest of the nuclei (Figure 5).

**Figure 6:**
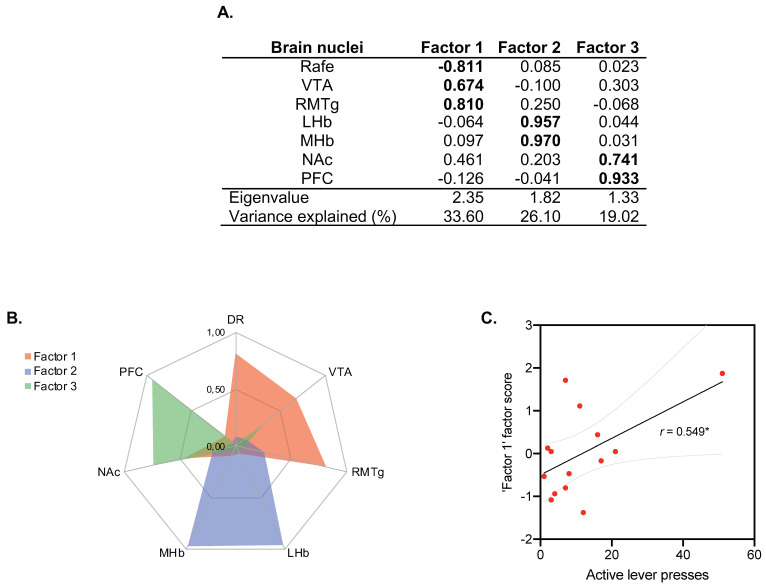
General regions extracted in principal component analyses. (**A**) Factors extracted from C-Fos data. A PCA revealed three independent dimensions indicative of the amount of activity in the Raphe/VTA/RMTg areas (Factor 1), the Hb (Factor 2) and the PFC/NAc regions (Factor 3). Numerical values indicate factor loadings. (**B**) Factor 1 was obtained from measures registered in DR, VTA and RMTg; Factor 2 was obtained from measures registered in LHb and MHb; Factor 3 was obtained from measures registered in NAc and CPF. A brain nucleus was considered to be included in a factor when its loading was >0.7 in absolute value (highlighted in bold). High factor scores indicate better performance in each dimension. (**C**) Correlation between behavioral data (pulsations to the active lever) of the ethanol self-administration test and C-Fos in Factor 1; r. = 0.549, * *p* = 0.042 (*n* = 14 animals/group).

The PCA revealed three independent factors representing the functional brain modules or networks underlying C-Fos IR that explained ~80% of the total variance (Figure 6A,B). The first factor encompassed DR, VTA and RMTg (33.60% of variance explained), the second factor was composed of LHb and mHb (26.10% of variance explained), while the third factor was composed of NAc and CPF (19.02% of variance explained). PCA statistical assumptions were satisfied, allowing its use and interpretation (KMO: 0.349; Bartlett’s sphericity test: X^2^(21) = 35.76, *p* = 0.023).

To determine the relevance of each brain network (factor) in the alcohol self-administration test, we tested for correlations of the factorial scores (FS) for each factor with the number of active lever presses in ethanol self-administration. The number of active lever presses could only be predicted by the scores in Factor 1, composed by DR (FS = −0.811)/VTA (FS = 0.674)/RMTg (FS = 0.810) (r = 0.549; *p* < 0.05; Figure 6C), indicating that rats that exhibited an increase in C-Fos IR in the VTA/RMTg circuitry were more prone to press the active lever in the alcohol self-administration test.

Additionally, immunohistochemistry of pCREB was performed in NAc. The administration of ESC (2.5 mg/kg) and the coadministration of ESC (2.5 mg/kg) and GAL(1–15) (0.3 nmol) did not show significant differences in the number of pCREB IR cells (Table S2).

## 4. Discussion

In the present study, we demonstrated that the combination of GAL(1–15) with ESC induced a substantial reduction of alcohol intake in the ethanol self-administration paradigm. Moreover, GAL(1–15) enhanced the reduction of reward capacity of ESC on different reinforcers such as sucrose or saccharine. The coadministration of GAL(1–15) + ESC significantly decreased the number of C-Fos-IR TH cell bodies in the VTA, and PCA analysis suggested that one functional network, including VTA, RMTg and DR, was involved in these effects on alcohol self-administration.

Interestingly in rats with alcohol consumption by self-administration, GAL(1–15) reversed the adverse ESC-mediated effects in depression-related behavioural test FST, confirming that the combination GAL(1–15) + ESC also improved the depressive symptoms induced by the ESC.

The operant self-administration models are a tool for studying reward-seeking motivated behaviour [36,37]. In this work, we used two operant self-administration models, alcohol and saccharin. In both tests, the combination of GAL(1–15) and ESC induced a substantial reduction in the number of reinforcements, suggesting that GAL(1–15) + ESC caused a loss of motivational behaviour induced by an artificial reinforcer. This effect in the loss of motivational behaviour is powerful since it is induced with the coadministration of threshold doses of GAL(1–15) and ESC [24,29].

Not only in the operant models but also in the non-operant model using the natural reinforcer sucrose (SPT) [38,39], the combination of GAL(1–15) and ESC induced a significant reduction in sucrose preference and sucrose intake, confirming a substantial effect for this cotreatment in the reward system.

The fact that the GAL(1–15) and ESC combination can modulate the reward system with both natural and artificial reinforcers such as alcohol, in addition to improving depressive symptoms in an animal model of depression such as OBX rats [29] opens up the possibility to use this combination as augmentation therapy in the depression and AUD comorbidity.

The role of neurotransmitter 5-HT as a modulator of reward function is widely described in the literature. Serotonin reduces the reinforcing properties of food, saccharin, alcohol, psychostimulants, and direct electrical brain stimulation [13,40,41,42,43]. Treatment with SSRIs or the 5-HT releaser dexfenfluramine consistently decreases instrumental responding for primary reinforcers such as food, drugs of abuse [44], and brain stimulation reward [45,46]. Moreover, both in SERT-KO mice and after acute administration of citalopram, the primary reinforcer saccharine is reduced [47]. Recent optogenetic studies also found that combining stimulation of DRN 5-HT neurons with a low dose of the SSRI citalopram reduced the operant responding by saccharine [40].

The mechanism involved in the 5-HT effect in the reward system affects the DA system. The serotoninergic neurons from DR innervate the VTA and inhibit mesolimbic DA activity [40]. Additionally, in the NAc, the MSNs neurons synapse onto DR 5-HT neurons, directly influencing reward processes [48,49]. Several studies showed that SSRIs, including ESC, reduced DA neuronal activity in the VTA and DA release in the striatum [50,51,52,53]. The reduction of DA neuronal activity is probably related to a serotonergic-dopaminergic interaction in the mesolimbic system via 5-HT2C receptors [53,54].

Our results are in agreement with these works since ESC reduced the reward capacity in the operant and non-operant models, confirming that 5-HT decreases the reward function. Moreover, the coadministration of GAL(1–15) + ESC produced a significant decrease in the number of C-Fos-IR TH cell bodies in the VTA, and the analysis of the relevance of the different brain networks in the alcohol self-administration test indicated that rats that exhibited an increase in C-Fos IR in the VTA/RMTg circuitry were more prone to press the active lever in the alcohol self-administration test, confirming the importance of VTA in the GAL(1–15) + ESC-mediated effect.

The dose of GAL(1–15) 0.3 nmol used was 10 times lower than the effective dose of GAL(1–15) in C-Fos immunohistochemistry [30,35], and GAL(1–15) 1 nmol lacked effect at the behavioural level [30] so no effect was expected. However, in future experiments, we will need to address a more detailed study of GAL(1–15) over C-Fos effects.

It is of high interest that the effect of GAL(1–15) and ESC coadministration was found in the FST, a depression behavioural test related with despair, in the animals with alcohol intake by self-administration. Our results indicated that ESC administration alone induced an increase in immobility and a decrease in swimming, suggesting a worsening of depressive symptoms. However, the coadministration of ESC with GAL(1–15) reversed the adverse effects induced by ESC alone in the FST.

ESC effects in naive rats show a variability of response: no effect [55], or a decrease [56] of immobility in the FST. Interestingly, chronic ethanol intake alters 5HT1A receptor density and/or expression in the brain [57,58,59], showing an increase in the density and expression of somatodendritic 5HT1A autoreceptor [57] and a 5HT1A autoreceptor supersensitivity [60] in the DR. This increase in the density and functional sensitivity of the 5HT1A autoreceptor could explain the pro-depressive effects of ESC in the FST; ESC through the SERT lock raises the central tone of 5-HT, which increases the activation-hypersensitive autoreceptors induced by the alcohol and produces a decrease in the 5-HT released. 

The interaction between GAL(1–15) and the 5HT1A receptor has been described both at the functional level and at the receptor level in recent years [22,26,27,28,61]. The existence of a 5HT1A-GALR1-GALR2 receptor mosaic would explain these results [26,62,63,64].

We have described the GAL(1–15) ability to modulate the 5HT1A receptor characteristics and expression level [26]. Thus, in the DR, GAL(1–15) reduced the density of 5HT1A autoreceptor and its mRNA levels, suggesting an enhancement in the firing rate of the ascending 5HT DR neurons [26]. This mechanism could underlie the enhancement of ESC by GAL(1–15) in the FST in the present work. GAL(1–15) could modulate the alcohol-induced increase in density and functional sensitivity at somatodendritic 5HT1A receptors in DR, thus avoiding the depressive adverse effects shown by ESC in alcohol-consuming rats. However, a detailed study of this mechanism should be carried out in the future.

In conclusion, our results indicate a potent effect of the combination GAL(1–15) with ESC in reducing the reward-seeking motivated by alcohol with a significant reduction of depressive adverse effects in rats. The results open up the possibility to use GAL(1–15) in combination with the SSRI Escitalopram as a novel strategy in AUD comorbidity with depression.

## Figures and Tables

**Table 1:** Combination of Escitalopram and Galanin (1–15) in the elevated plus maze and the open field test.

Treatment	EPM		OFT	
	% Entries in Open Arms	% Time in Open Arms	N° Entries	Time Entries(s)
Saline + aCSF	23.6 ± 8.9	14.9 ± 7.4	23.6 ± 4.0	62.0 ± 15.3
ESC 2.5 mg/kg + aCSF	18.9 ± 8.2	14.8 ± 8.0	25.9 ± 4.3	59.0 ± 12.0
ESC 2.5 mg/kg + GAL(1–15) 0.3 nmol	7.9 ± 2.8	1.9 ± 0.7	22.1 ± 4.3	36.4 ± 10.2

Effect of the administration of Galanin (1–15) [GAL(1–15)] and Escitalopram (ESC) in the elevated plus maze (EPM) and the open field test (OFT). ESC was administrated intraperitoneal (ip) 23, 5 and 1 h before the test, and GAL(1–15) 0.3 nmol or artificial cerebrospinal fluid (aCSF) was administered i.c.v 15 min before the test. Saline + aCSF injected rats were used as control group (*n* = 7–8 animals/group). Data represent mean ± standard error of the mean of % entries and % time in open arms of EPM and number of entries and time entries in center of the OFT. There were no differences according to a one-way analysis of variance (ANOVA) between the experimental groups.

## Data Availability

The data presented in this study will be openly available in RIUMA-University of Malaga once the manuscript is accepted for publication.

## Supplementary Materials

The following supporting information can be downloaded at: https://www.mdpi.com/article/10.3390/biomedicines10020412/s1. Figure S1. Effect of the administration of GAL(1–15) and ESC in the saccharin self-administration test; Table S1. ESC dose response curve in the sucrose preference test (SPT); Table S2. Effect of the administration of GAL(1–15) and ESC on pCREB expression.
