# Supplementary material for: The Combination of Galanin (1–15) and Escitalopram in Rats Suggests a New Strategy for Alcohol Use Disorder Comorbidity with Depression
Source: Biomedicines. 2022 Feb 9;10(2):412. doi: 10.3390/biomedicines10020412 (PMC8962322; doi:10.3390/biomedicines10020412)
Supplement: Supplementary file 1 [file biomedicines-10-00412-s001.zip › biomedicines-1516714-supplementary.pdf]

SUPPLEMENTARY MATERIAL AND METHODS

Stereotaxic surgery

This protocol has been described previously (Diaz-Cabiale et al. 2011; Millon et al. 2015). Briefly, the rats were anesthetized with isoflurane (ISOFLO® ZOETIS ABOTT Liquid gaseous anaesthetic,100% Isoflurane) 5% in induction chamber and maintenance of 1.5-2.5% Isoflurane mask, and placed in the stereotactic instrument. A chronic 22-gauge stainless-steel guide cannula was stereotaxically implanted into the right lateral cerebral ventricle (+1.4 mm lateral, -1 mm posterior to bregma, and 3.6 mm below the surface of the skull) (Paxinos, 1986). Animals were individually housed after surgery and had a recovery period of 7 days prior to behavioural test.

Administration of substances and drugs

In the experimental groups we used the following drugs: Escitalopram Oxalate (Sigma-Aldrich, San Luis, EEUU) and GAL(1–15) (TOCRIS, Bristol, United Kingdom). GAL(1-15) or artificial cerebrospinal fluid (aCSF) was injected Intracerebroventricular (i.c.v) 15 min before the test. aCSF composition is 120nM NaCl, 20nM NaH_2CO_3, 2nM KCl, 0.5nM KH_2PO_4, 1.2nM $CaCl_2$, 1.8 nM $MgCl_2$, 0.5 nM Na_2SO_4, and 5.8 nM Dglucose, pH 7.4.

A subchronic injection pattern of escitalopram (ESC) was followed. ESC was injected intraperitoneal (ip) 23, 5 and 1 hour before the test. For intraperitoneal (ip) administration of ESC solutions were dissolved in 0.9% saline solution and were administered in a volume of 0.2 ml/100 g body weight. The doses employed and the injection protocol were based on those of previous studies (Millon et al., 2017, 2019; García-Durán et al; 2021).

Intracerebroventricular Injections

The injections in the lateral ventricle were performed using a 26-gauge stain lesssteel injection cannula connected via a PE-10 tubing to a Hamilton syringe. The total volume was 5 µl per injection and the infusion time was 1min with special care that during the process the dilutions were administered fluently and without overflowing. Solutions were prepared freshly and the peptides were dissolved in aCSF.

Operant self-administration apparatus and training methods

Training and testing were conducted in standard operant chambers (Panlab, Barcelona, Spain). Each chamber was equipped with a drinking reservoir (0.10 ml) positioned 4 cm above the grid floor in the center of the front panel of the chamber, and two retractable levers were located 3 cm to right an left of the dinking receptacle.One lever was paired with the delivery of saccharin or ethanol as a reward (active lever), whereas the other lever was paired with no reward (inactive lever). Lever pressing was paired with a conditioned stimulus light adjacent to the lever was illuminated when the FR 1 requirement was met and signalled to the rat that fluid had been dispensed as a reward. Chambers were connected to a computer running Packwin Sofware (2.0, Panlab, Barcelona, Spain) which was used to automatically record the number of active and inactive lever responses and the number of reinforcement for each rat in each session (Wilson et al., 2018).

Ethanol self-administration test

The self- administration behaviour was induced by a two – step paradigm: first, rats were exposed to intermittent access to 10 % ethanol and 0.2 % saccharin for 3 weeks to facilitate the acquisition of a high level of ethanol intake (Lebourgeois et al; 2018) with minors modifications. Rats were given access to two bottles, one bottle containing tap water and the other containning 10 % ethanol and 0.2 p% saccharin, for 24 hour sessions on Mondays, Wednesdays and Fridays for 3 weeks (a total of nine drinking sessions). At the end of each session, bottles were weighted to assess both ethanol consumption (g ethanol/Kg body weight) and preference (the ratio of ethanol consumed to total fluid intake). The bottle placement in the cage (left or right) was alternated between each session to avoid side preferences. Before self-administration, rats were placed on a water restriction schedule for 2–4 days to facilitate training of lever pressing. The rats were initially trained to self-administer 10% ethanol and 0.2% saccharin, decreasing the saccharin percentage over the days and maintaining that of alcohol finally getting the rats to consume only 10% ethanol. Rats were trained to self-administer a 10 % ethanol solution during short session operant access period (30 minutes), 5 days a week, using a fixed ratio 1 (FR1). Operant chambers were individually housed with a lever on each side that was linked to a

reservoir to dispense fluid into a receptacle. Each reservoir was calibrated to dispense 0.1mL when the fixed ratio of lever presses was obtained.

Elevated Plus Maze

The maze is 40 cm above the floor, has cross shaped platforms with two open arms with 2 cm high pleixglass sides, and two closed arms with 40 cm high opaque walls. Rats were placed on the central platform facing an open arm and allowed to explore the maze for 5 min following 30 min acclimation to a room with dim light. Entry into the open (OA) or closed (CA) arm was defined as entering with all four paws. Anxiety index was calculated as: 1- [(time spent in OA/total time on the maze)/2 + (number of entries to the OA/total number of entries into OA and CA)/2]. Analysis of all behavioral measurements were performed by individuals blinded to the experimental groups. After each trial, all surfaces were cleaned with a paper towel and 70% ethanol solution.

C-Fos immunohistochemistry

The sections were processed free floating. Endogenous peroxidase activity was removed by incubating the sections with 10% H_2O_2 and 10% methanol for 30 min. After blocking and permeabilization with 0.2% Bovine Serum Albumin (Sigma) with 0.5% Triton X-100 (10 min, room temperature), the sections were incubated with a mouse polyclonal antibody (Santa Cruz Biotech, sc-271243) raised against the C-Fos protein (1/200). Overnight incubation at room temperature was performed in 0.01 M PBS containing 0.5% Triton X-100. The sections were washed 3 times in PBS and incubated with biotinylated anti-mouse polyclonal goat (Dako E 0433) (1/600) for 1 h at room temperature. The immunostaining was performed with streptavidin peroxidase (SIGMA E2886) (1/1000) for 1 h at room temperature. The chromogen used was 0.05% 3-30-diaminobenzidine tetrahydrochloride (DAB) (Sigma, Spain) intensified with nickel chloride hexahydrate (Sigma, Spain) 0.04% (w/v), giving darker black nuclei staining.

Sections from every animal from each experimental group were processed simultaneously. Omission of the primary antibody resulted in no detectable staining. After mounting the sections on gelatin-chromium coated slides, the sections were dehydrated and coverslipped with DPX (Panreac, Barcelona,

Spain). Thus, every section was numbered according to the rostrocaudal level (Paxinos, 1986).

Images are taken with an Olympus VS120 microscope with a PLAN AP 10x objective. The areas of interest are manually cut out, and a Gaussian filter is applied to them. A Find_Maxima function that locates the C-Fos marks is used to the resulting image with the FIJI program (Schindelin et al., 2012).

C-Fos/5HT immunohistochemistry

A double immunohistochemistry of 5-hydroxytryptamine (5-HT) and C-Fos and was performed to study specific cell activation in dorsal raphe (DR).
5-HT immunostaining was performed under the same conditions as C-Fos but using 0.9% saline Tris buffer 0.1 M (pH 7.6) instead of saline phosphate buffer. The primary antibody was a rabbit monoclonal antibody raised against 5-HT (20080, INCSTAR) (1/20000). Nickel chloride was not added to the chromogen solution in the second incubation for immunostaining in order to get a brownish reaction. Sections from every animal from each experimental group were processed simultaneously. Omission of the primary antibody resulted in no detectable staining. After mounting the sections on gelatin-chromalum coated slides, the sections were dehydrated and coverslipped with DPX (Panreac, Barcelona, Spain). Thus, every section was numbered according to the rostrocaudal level (Paxinos, 1986).

Images are taken with Olympus VS120 microscope with a PLAN AP 10x objective. The areas of interest are manually cut out and manually counted C-Fos and 5HT marks with the cell counter, tool of the FIJI program, (Schindelin et al., 2012).

C-Fos/TH immunohistochemistry

A double immunohistochemistry of tyrosine hydroxylase (TH) and C-Fos was performed to study specific cell activation in the ventral tegmental área (VTA). TH immunostaining was performed under the same conditions as C-Fos. The primary antibody was a mouse monoclonal antibody raised against TH (T1299,

Sigma) (1/2500). Nickel chloride was not added to the chromogen solution in the second incubation for immunostaining to get a brownish reaction. Sections from every animal from each experimental group were processed simultaneously. Omission of the primary antibody resulted in no detectable staining. After mounting the sections on gelatin-chromalum coated slides, the sections were dehydrated and coverslipped with DPX (Panreac, Barcelona, Spain). Thus, every section was numbered according to the rostrocaudal level (Paxinos, 1986).

Images are taken with Olympus VS120 microscope with a PLAN AP 10x objective. The areas of interest are manually cut out and manually counted C-Fos and TH marks with the cell counter, tool of the FIJI program, (Schindelin et al., 2012).

pCREB immunohistochemistry

After the selection of sections, the recovery of Epitopes is carried out: passage of plate tissue (Hoffman) to tubes with citrate buffer (10mM Citric Acid in H_2Od pH6) in a bath at 70ºC 30´. Endogenous peroxidase activity was removed by incubating the sections with 10% H_2O_2 and 10% methanol for 30 min. After blocking and permeabilization with 0.2% Bovine Serum Albumin (Sigma) with 0.5% Triton X-100 (10 min, room temperature), the sections were incubated with a rabbit polyclonal antibody (Milipore 06-519) raised against the pCREB protein (1/500). Overnight incubation at room temperature was performed in 0.01 M PBS containing 0.5% Triton X-100. The sections were washed 3 times in PBS and incubated with biotinylated anti-rabbit polyclonal (Amersham, RPN 1004) (1/200) for 1 h at room temperature. The immunostaining was performed with streptavidin peroxidase (SIGMA E2886) (1/1000) for 1 h at room temperature. The chromogen used was 0.05% 3-30-diaminobenzidine tetrahydrochloride (DAB) (Sigma) intensified with nickel chloride hexahydrate (Sigma) 0.04% (w/v), giving darker black nuclei staining.

Sections from every animal from each experimental group were processed simultaneously. Omission of the primary antibody resulted in no detectable staining. After mounting the sections on gelatin-chromium coated slides, the sections were dehydrated and coverslipped with DPX (Panreac, Barcelona,

Spain). Thus, every section was numbered according to the rostrocaudal level (Paxinos, 1986).

Images are taken with an Olympus VS120 microscope with a PLAN AP 10x objective. The areas of interest are manually cut out, and a Gaussian filter is applied to them. A Find_Maxima function that locates the pCREB marks is used to the resulting image with the FIJI program (Schindelin et al., 2012).

Table S1. Escitalopram (ESC) dose response curve in the sucrose preference test (SPT). ESC was administrated intraperitoneal (ip) 23, 5 and 1 hour before the test. Saline injected rats were used as control group. (n= 7-12 animals/group). Data represents mean ± standard error of sucrose intake (gr/Kg) and sucrose preference (%). ** p<0.01 vs Saline; #p<0.05 vs saline, ## p<0.01 vs ESC 7.5mg/Kg, according to a one-way analysis of variance (ANOVA) followed by Fisher’s least significance difference test.

**Treatment**	***Sucrose Intake (gr/Kg)***	***Sucrose Preference (%)***
**Saline**	0.4±0.04	78.2±4.8
**ESC 7.5 mg/Kg**	0.2±0.1**	62.7±5.7#
**ESC 10 mg/Kg**	0.4±0.05	84.2±3.1##

A.

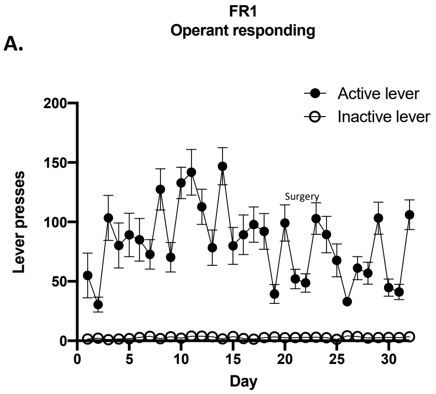

B.

Percent Baseline (FR1)			
Treatment	Nº reinforcements	Active lever presses	Inactive lever presess
Saline	203.6±34.2	197.3±33.7	90.2±20.6
ESC 2.5mg/Kg	123.4±37.8	119.8±37.6	100.7±38.9
ESC 5mg/Kg	80.2±27.7*	74.8±25.0*	45.9±17.7
ESC 7.5mg/kg	51.3±10.6***	50.9±10.5***	16.9±6.2

C.

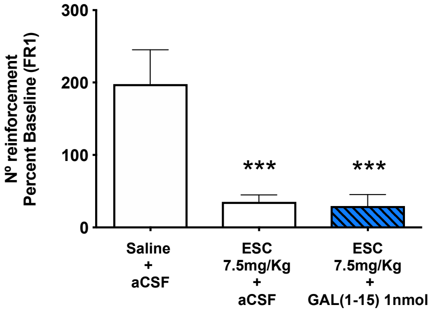

D.

Percent Baseline (FR1)		
Treatment	Active lever presses	Inactive lever presess
Saline + aCSF	194.9±48.8	36.5±13.4
ESC 7.5mg/Kg + aCSF	35.5±10.1**	9.7±5.4*
ESC 7.5mg/Kg + GAL(1-15) 1nmol	28.6±15.7**	0±0**

Figure S1. Effect of the administration of Galanin (1-15) [GAL(1-15)] and Escitalopram (ESC) in the saccharin self-administration test. ESC was administrated intraperitoneal (ip) 23, 5 and 1 hour before the test and GAL(1-15) 1 nmol or artificial cerebrospinal fluid (aCSF) were administered icv 15 min before the test. Saline+ CSF injected rats were used as control group **A.** Animals displayed a consistent preference for 0.2% (w/v) saccharin (active lever) over no reward (inactive lever) during the FR1 operant responding phase. **B.** Data represents a mean ± standard error of the mean of the number of saccharin reinforcements, active lever and inactive lever presses according to Percent Baseline in FR1 during the test period. * $p<0.05$ vs saline; *** $p<0.001$ vs saline according to a one-way analysis of variance (ANOVA) followed by Fisher's least significance difference test. (n=8-14 animals/group). **C**. Vertical bars represent a mean ± standard error of the mean of the number of saccharin reinforcements and active lever presses according to Percent Baseline in FR1 during the test period. ***$p<0.001$ vs saline, according to a one-way analysis of variance (ANOVA) followed by Fisher's least significance difference test. (n=7-8 animals/group). **D**. Data represents a mean ± standard error of the mean of the active lever and inactive lever presses according to Percent Baseline in FR1 during the test period. *$p<0.05$ vs saline; ** $p<0.01$ vs saline, according to a one-way analysis of variance (ANOVA) followed by Fisher's least significance difference test.

Table S2. Effect of the administration of Galanin (1-15) [GAL(1-15)] and Escitalopram (ESC) on pCREB expression. ESC was administrated intraperitoneal (ip) 23, 5 and 1 hour before the test and GAL(1-15) 0.3nmol or artificial cerebrospinal fluid (aCSF) were administered i.c.v 15 min before the test. Saline + aCSF injected rats were used as control group. (n=4-5 animals/group). Data represents mean ± standard error of pCREB cells/mm^2 in NAc. There are no differences according to a one-way analysis of variance (ANOVA) between the experimental groups.

Treatment	NAc (pCREB cells/mm^2)
Saline + aCSF	1654±175.0
ESC 2.5mg/Kg + aCSF	2092±698.7
ESC 2.5mg/Kg + GAL(1-15) 0.3nmol	1588±179.0
